# A socio-ecological analysis of parents’ experiences of stillbirth in Limpopo, South Africa

**DOI:** 10.4102/safp.v67i1.6119

**Published:** 2025-10-16

**Authors:** Lunghile Shivambo, Dumile Gumede

**Affiliations:** 1 Department of Development Studies, College of Human Studies, University of South Africa, Pretoria, South Africa; 2 Executive Deans Office, Faculty of Health Sciences, Durban University of Technology, Durban, South Africa

**Keywords:** stillbirth, parents, socio-ecological framework, healthcare communication, stigma, healthcare

## Abstract

**Background:**

Stillbirth is a profoundly personal experience shaped by sociocultural norms, healthcare systems, and policies. Limited research exists on parents’ lived experiences in South Africa. This study explored socio-ecological factors influencing parents’ experiences of stillbirth in Limpopo, South Africa.

**Methods:**

This qualitative exploratory study used in-depth interviews with 12 purposively selected parents. Interviews were conducted in Xitsonga, transcribed, translated, and thematically analysed using ATLAS.ti.

**Results:**

The findings revealed that parents’ experiences of stillbirth were shaped by a range of interconnected factors across multiple levels of the socio-ecological framework. At the individual level, participants expressed varying understandings of stillbirth, emotional distress, self-doubt and fear of recurrence. Interpersonal relationships played a dual role, with some participants receiving strong emotional support from partners and family members, while others experiencing blame and stigma. Organisational factors included positive and negative experiences with religious institutions and healthcare services, with reports of compassionate care and critical gaps in communication and response time. Societal influences were reflected in cultural beliefs, such as *Xirheti*, which shaped perceptions of repeated stillbirths and contributed to stigma. At the policy level, participants not only recognised the importance of antenatal care as promoted by national guidelines but also highlighted systemic barriers such as delayed transport and limited access to timely healthcare services.

**Conclusion:**

Addressing the impact of stillbirth requires a multi-level approach that integrates personal, social, cultural, healthcare, and policy dimensions to support parents.

**Contribution:**

This study offers evidence to inform more family-centred and system-responsive approaches in primary and maternal healthcare.

## Introduction

Stillbirth remains a significant public health issue globally, with profound emotional, psychological and social impacts on parents and families.^1^ Stillbirth refers to the birth of an infant showing no signs of life after 28 completed weeks of gestation.^2^ Globally, in 2019, an estimated 2 million babies were stillborn, with sub-Saharan Africa (SSA) accounting for nearly 50% of the global burden of stillbirths.^3,4^ In South Africa, the number of stillbirths has risen sharply over the years, reaching 15 908 cases in 2020.^5^ Stillbirth can result from a range of causes, including intrapartum complications; prolonged pregnancy; maternal infections such as malaria, syphilis and human immunodeficiency virus (HIV); underlying maternal health conditions such as hypertension, obesity and diabetes mellitus; and foetal growth restriction (FGR) and congenital abnormalities.^6,7^ Stillbirth is widely recognised as one of the most devastating and traumatic life events, often leading to enduring negative consequences for parents, including mental health challenges and disruptions in relationships.^8^ Despite advances in maternal and neonatal healthcare, the burden of stillbirth persists, particularly in settings where healthcare access and quality are limited. Very few studies have addressed the experiences of parents after stillbirth in South Africa. Understanding the experiences of parents who experienced a stillbirth is critical to developing responsive and culturally appropriate support systems and interventions that address their unique emotional, psychological and social needs.

Parents’ experiences of stillbirth are shaped by a multitude of factors, including cultural beliefs, healthcare access and interpersonal dynamics.^9,10^ Both cultural and religious interpretations of stillbirth often influence how individuals experience stillbirth.^11^ For instance, stillbirth may be attributed to witchcraft or personal behaviours, which can lead to stigmatisation. Furthermore, healthcare systems often fail to provide adequate support to affected individuals following stillbirth, with communication gaps and delays in medical attention exacerbating their distress.^12^ Spousal and family support have been identified as critical in mitigating the psychological toll of stillbirth, but the availability and quality of this support vary widely.^6^

Stillbirth is a devastating event with far-reaching implications for parents, families and communities.^13^ The literature highlights several key themes relevant to understanding the socio-ecological factors shaping parents’ experiences of stillbirth, ranging from cultural interpretations and emotional responses to systemic and policy-level barriers.

Cultural interpretations play a significant role in shaping how stillbirth is perceived and processed.^14^ In many societies, stillbirth is attributed to spiritual or supernatural causes, such as witchcraft, which can influence grieving processes and coping mechanisms.^15,16^ For instance, women in African contexts often face stigma and self-blame because of cultural norms that associate stillbirth with personal failure or familial curses.^17^ Despite the recognition that cultural interpretations significantly shape how stillbirth is perceived and processed, there remains a limited understanding of the specific cultural, social and systemic factors influencing the experiences of parents in the South African context. South Africa is a culturally diverse country with varied beliefs, traditions and healthcare access levels across different communities.

The emotional toll of stillbirth is profound, with parents often experiencing grief, depression, anxiety and post-traumatic stress disorder.^18^ Fathers, in particular, struggle with feelings of helplessness and inadequacy as they navigate their grief while supporting their partners.^19,20,21^ Repeated stillbirths exacerbate these emotions, leading to a heightened fear of recurrence and diminished self-concept.^22^ Fathers may resort to violent behaviour towards their partners, particularly when influenced by substance use. A systematic study examining the psychological impact of stillbirth on parents revealed that both mothers and fathers experience unique emotional and psychological distress. Mothers commonly struggle with feelings of depression, anxiety, guilt, anger and shame. These emotions are often compounded by a profound sense of confusion, emptiness, personal failure and a disconnection or rejection of their bodies^23^ Fathers, while experiencing similar emotions, often express their grief differently. They may cope by returning to work earlier than expected and may be more prone to increased substance use as a way of managing their emotional distress.^20^ Examining the socio-ecological factors shaping the experiences of stillbirth is essential to gaining a comprehensive understanding of how various interconnected influences affect parents’ experiences of stillbirth in the South African context.

Interpersonal relationships significantly influence how parents experience stillbirth. Spousal support has been identified as a critical factor in mitigating emotional distress, fostering resilience and strengthening marital bonds.^24^ However, some parents face stigma from family members, which can be a negative experience of stillbirth.^15^ A study conducted in rural Pakistan revealed that some women faced blame and social stigma following a second stillbirth.^15^ A scoping review study further supports these findings, highlighting that parents who experience stillbirth often encounter various forms of stigma, including public stigma.^25^ This form of stigma manifests through discrimination, unkind treatment, social avoidance and even ridicule directed at bereaved parents because of their loss. Despite the growing global awareness of the psychological and social impact of stillbirth, there remains a significant gap in knowledge regarding how parents in the South African context experience and navigate this loss. Specifically, there is limited research exploring the sociocultural, emotional and systemic factors that shape their experiences regarding grieving processes, coping mechanisms and access to support services within the country’s diverse and complex socio-economic landscape.

The role of healthcare systems in addressing stillbirth is critical yet often fraught with challenges. Delays in medical attention, inadequate communication and a lack of emotional support from healthcare providers have been widely reported.^26^ Positive interactions with healthcare providers, such as clear communication about the causes of stillbirth and respectful handling of the body of a stillborn baby, are crucial for facilitating closure and reducing emotional distress.^27^ Additionally, religious institutions often complement healthcare services by providing spiritual support,^28^ highlighting the importance of integrated care models. Despite global evidence highlighting the critical role of healthcare systems and religious institutions in supporting parents after stillbirth, significant knowledge gaps exist within the South African context. South Africa’s diverse sociocultural landscape, coupled with disparities in healthcare access and quality, presents unique challenges and experiences that remain underexplored.

At the societal level, social networks and religious organisations play a vital role in supporting bereaved parents following stillbirth.^9^ Evidence shows that reassurance from church members fostered hope for the future,^29^ while practices such as reading spiritual texts, praying, making offerings and lighting temple lamps provided a sense of purpose and comfort after the loss.^11^ While churches and religious communities can offer important sources of comfort and support, they may also contribute to distress, especially when parents encounter judgement, stigma or theological interpretations that exacerbate their grief. A qualitative meta-synthesis found that religious beliefs restricting parents from seeing, touching or participating in funeral rites for their stillborn baby were often questioned and contributed to ongoing distress.^30^ This highlights the dual role of religious institutions as both sources of support and potential harm for parents experiencing stillbirth. Furthermore, community-based interventions, together with healthcare facilities, have been shown to reduce stigma and improve access to care.^31^ Policy-level factors, including South African antenatal care guidelines and healthcare infrastructure, also shape parents’ experiences. Ensuring equitable access to quality antenatal care and addressing systemic barriers, such as understaffing and resource constraints, are critical for reducing stillbirth rates and improving outcomes.^32^ Despite the acknowledgement that community and policy-level factors significantly influence parents’ experiences following stillbirth, critical gaps remain in the South African context regarding how these elements interact and shape stillbirth experiences. South Africa’s disparities in healthcare access introduce complexities that are not yet fully understood.

The existing literature highlights the multifaceted nature of stillbirth and its profound impact on parents, highlighting the interplay of cultural beliefs, emotional responses, interpersonal dynamics, healthcare systems and policy-level factors in shaping their experiences.^33^ However, despite increasing awareness of the complexities surrounding stillbirth, significant research gaps persist, particularly in low-resource settings such as South Africa. There remains a limited understanding of the lived experiences of parents following stillbirth within the unique sociocultural and healthcare contexts of the country.

Understanding the experiences of parents following stillbirth is essential for developing effective, contextually relevant interventions and policies. Despite growing awareness of the emotional and psychological impact of stillbirth, limited research has examined how multi-level socio-ecological factors, spanning the individual, interpersonal, societal, organisational and policy levels, shape parents’ perceptions and experiences of stillbirth.

This study addresses that gap by exploring the socio-ecological factors influencing parents’ experiences of stillbirth in South Africa, to inform targeted interventions and policy recommendations to better support parents and improve maternal healthcare services.

Guided by the socio-ecological framework, originally developed from Bronfenbrenner’s ecological systems theory, this study recognises that individual experiences are shaped by a dynamic interaction of personal, social and systemic influences.^34^ This framework is widely used in public health and is particularly suited to examining complex issues such as stillbirth, where cultural beliefs, healthcare practices and structural barriers converge.^35^

By applying this framework, the study offers a holistic understanding of stillbirth as both a personal and socially embedded experience, providing insights into how various levels of influence intersect and identifying opportunities for more responsive and culturally appropriate care.

## Research methods and design

### Study design and setting

This study employed a qualitative exploratory design to explore the socio-ecological factors influencing parents’ experiences of stillbirth. A qualitative design was chosen for its ability to provide an in-depth understanding of a complex phenomenon within its real-life context.^36^ This design is particularly suitable for examining the multilayered influences on parents’ experiences, as framed by the socio-ecological framework. The study was conducted at Muyexe village in the Mopani district within Limpopo province in South Africa. The village is densely populated with Xitsonga ethnic individuals, providing an opportunity to explore how cultural beliefs, traditions and social norms within this specific ethnic community influence perceptions and experiences of stillbirth. Between 01 February 2018 and 31 October 2021, a total of 392 stillbirths were recorded at Kgapane Hospital in the Mopani district,^37^ highlighting that stillbirths are prevalent in the province.

### Population and sampling

The study population included parents (women and men) who were 18-years-old and above and had experienced a stillbirth. Participants were selected through purposive and snowball sampling strategies to ensure that the sample was representative of individuals with lived experiences of stillbirth. Inclusion criteria were mothers and fathers aged 18-years-old or older; individuals who had experienced at least one stillbirth within the past five years; and participants who were willing to provide informed consent and participate in the study.

Recruitment was facilitated by a local home-based care worker and through community leaders. By involving a local home-based care worker and community leaders, the recruitment approach effectively ensured access to individuals who met the study criteria by leveraging trust, cultural knowledge and targeted outreach. This approach not only increased participation but also contributed to the study’s ethical integrity and relevance by ensuring that the voices of those directly affected by stillbirth were represented.

### Data collection

Data collection was conducted by a Master’s Degree student (L.S.) who was trained in qualitative research methods and had prior experience in maternal and child health. There was no prior relationship between the interviewer and participants. Efforts were made to build rapport and trust at the beginning of each interview. The data collection process was supervised by an experienced qualitative researcher (D.G.), who provided ongoing guidance and support throughout and held regular debriefing sessions with L.S. to ensure methodological rigour and ethical adherence.

Data were collected through in-depth interviews, allowing participants to narrate their experiences in their own words. Interviews were conducted with participants who met the inclusion criteria of having experienced a stillbirth within the past five years. The interview guide was developed, based on the socio-ecological framework, to allow for flexibility in probing emerging topics. It included questions addressing personal perceptions and emotions surrounding stillbirth, interpersonal relationships, including family and spousal support, experiences with healthcare providers and community organisations, cultural and societal influences on stillbirth experience, and awareness of policies and healthcare practices related to stillbirth prevention. Interviews were conducted in the participants’ preferred language, Xitsonga, to ensure comfort and clarity, and they were conducted in a setting chosen by participants to ensure privacy and confidentiality. Each interview lasted approximately 45 min to 60 min and was audio-recorded with participants’ consent. The interviews took place between August and September 2023. Field notes were also taken to document non-verbal cues and contextual details. Interviews continued until no new themes or significant variations in responses emerged, indicating thematic saturation. Saturation was assessed iteratively during data analysis, as two researchers independently reviewed transcripts and compared emerging codes. By the eleventh and twelfth interviews, recurring patterns were consistently identified, and no new categories of information were observed, suggesting that additional interviews were unlikely to yield substantially new insights. This informed the authors’ decision to conclude data collection at 12 participants.

### Data analysis

Thematic analysis was used to analyse the data, following the six-step framework of Braun and Clarke.^38^ The six steps are familiarisation, coding, generating themes, reviewing themes, defining themes and writing the report. Data were transcribed verbatim and translated into English. ATLAS.ti version 23 software was employed to facilitate the organisation and coding of data. Both authors read through the transcripts several times to identify concepts. All the concepts were coded and arranged into categories. Both authors individually organised these concepts into main themes and sub-themes. A consensus was reached between the authors. Themes were grounded in the data and supported by direct participant quotes to ensure transparency and credibility. Field notes were used to complement the interview transcripts by capturing non-verbal cues, contextual observations and the emotional tone of participants during data collection. These notes enriched the analysis by providing additional insights into participants’ experiences, helping to interpret ambiguous responses and identify underlying emotions or cultural nuances that may not have been fully expressed in the transcripts alone. The socio-ecological framework guided the analysis, ensuring that themes reflected factors at individual, interpersonal, organisational, societal and policy levels. The authors applied a 32-item checklist, the Consolidated Criteria for Reporting Qualitative Research (COREQ), to ensure comprehensive reporting of qualitative data.^39^

### Trustworthiness

To enhance the credibility, transferability, dependability and confirmability of the study, multiple measures were implemented. Triangulation was employed by utilising various data sources, including interviews and field notes, to ensure a comprehensive understanding of the phenomenon. Researchers practised reflexivity throughout the study, acknowledging how their backgrounds, beliefs and experiences could influence data collection and interpretation. Reflexive journaling and regular team debriefings were used to support this process. Moreover, thick descriptions of participants’ experiences and the study context were provided to support the transferability of findings to similar settings or populations.

### Ethical considerations

Ethical approval for the study was obtained from the institutional research ethics committee (Reference: 64079228_CREC_CHS_2023). Participants were provided with detailed information about the study, including its objectives, potential risks and benefits. Written informed consent was obtained from all participants. Confidentiality was ensured by anonymising data and securely storing all research materials. Participants were also informed of their right to withdraw from the study at any time without consequences. To mitigate any potential discomfort related to the research topic, participants were provided with information about the available psychosocial support services through a local social worker.

## Results

### Characteristics of study participants

A total of 12 participants (eight mothers and four fathers) were included in the study, with ages ranging from 23-years-old to 61-years-old. Most participants reported experiencing more than one stillbirth, which highlights the profound emotional and psychological impact of repeated loss. Seven participants were married, and five participants were unmarried, including two couples. Participants’ educational backgrounds varied, with the lowest level being Grade 2 and the highest being Grade 12. However, all participants had received some form of formal education.

### Factors influencing parents’ experiences of stillbirth

The experiences of parents following stillbirth are shaped by a complex interplay of factors that operate across various levels of influence. These factors were categorised using the socio-ecological framework, encompassing individual, interpersonal, organisational, societal and policy levels. Each level provides unique insights into the multifaceted nature of stillbirth, from personal emotional responses to systemic healthcare challenges and societal norms. This approach highlights the interconnectedness of these factors and their combined impact on how parents perceive and experience stillbirth. Results are presented with illustrative quotes, and variations in experiences are highlighted to reflect the complexity and diversity of responses.

### Individual level factors

At the individual level, four themes emerged, namely, understanding of stillbirth, emotional responses, self-doubt and shame, and fear of recurrence, highlighting key personal factors shaping parents’ perceptions and experiences.

#### Understanding of stillbirth

The results reveal that participants held varied understandings of stillbirth, both in how they defined the condition and in what they believed to be its causes.

In defining stillbirth, participants commonly understood it as the birth of a baby showing no signs of life. One participant stated:

‘It is a baby that is born already dead … that baby is born with no life at all.’ (P11, male, 24-years-old)

Consistent with this understanding, another participant said:

‘It’s a baby that dies before birth.’ (P12, male, 54-years-old)

Regarding the causes of stillbirth, while many participants described it as the birth of a lifeless baby, their understanding of its underlying causes varied. Some participants interpreted stillbirth through a supernatural lens, with one perspective attributing the loss to the will of God, viewing it as a divinely ordained event beyond human control. This was illustrated by two excerpts:

‘God did His own will.’ (P06, female, 47-years-old)

‘I attribute everything to the will of God. I believe He is the one who knows everything. He created that baby, and if He decided to take the baby before it could even breathe or set foot on this earth, then that’s His decision. It’s okay, because no matter how much I try to crack my brain thinking about what might have happened, it won’t change anything. I just have to accept what happened, the way it happened.’ (P07, male, 61-years-old).

Both participants believed that the baby’s death was part of God’s divine plan; however, P07 chose not to dwell on the cause of the stillbirth, recognising that seeking explanations would not alter the outcome.

In contrast to viewing stillbirth as an act of divine will, some participants attributed the cause to witchcraft, a belief rooted in supernatural explanations. This perspective is reflected in the following excerpts:

‘I was always thinking about my baby and how I lost my baby. I even thought that maybe witchcraft was involved. Although I didn’t know who might have bewitched my child, I believed that someone had done something. At the hospital, they didn’t give many details, so I truly felt that witchcraft might have played a role.’ (P04, female, 39-years-old)

‘When it happened, I even thought that witchcraft was involved. We are Africans, and witchcraft is always there.’ (P09, male, 38-years-old)

Both participants acknowledged the possibility of witchcraft as a cause of stillbirth. However, there is a notable difference in how they engage with this belief. P04 openly explores the idea of witchcraft, expressing uncertainty but still leaning towards it as a plausible explanation because of the lack of clarity provided by the hospital. P09, on the contrary, acknowledges witchcraft as part of African cultural reality.

In addition, some participants associated stillbirth with lifestyle-related factors, including excessive stress during pregnancy, poor adherence to antenatal care, heavy physical labour during pregnancy, alcohol consumption during pregnancy and use of over-the-counter medication and traditional medicine.

Firstly, excessive emotional stress during pregnancy emerged as a perceived cause of stillbirth among some participants. One participant highlighted this concern, stating:

‘Stillbirth happens when you stress too much while pregnant.’ (P06, female, 47-years-old)

When probing about the sources of excessive stress during pregnancy, it was attributed to marital conflicts or rejection by a partner. P06 gave an example of a marital conflict, although not drawing from her personal experience, which may lead to negative pregnancy outcomes:

‘It usually happens, for example, when the father rejects the pregnancy and wants nothing to do with the baby. Naturally, a person would feel stressed in such a situation.’ (P06, female, 47-years-old)

Secondly, poor adherence to antenatal care was attributed to stillbirth, believing that missed check-ups or delayed visits may have contributed to complications. As one male participant, who had experienced stillbirths twice, stated:

‘Sometimes as parents, we ignore the importance of going for antenatal care … skipping antenatal care is a problem.’ (P07, male, 61-years-old)

While the participant recognised the importance of antenatal care in promoting a healthy pregnancy, they did not elaborate on the reasons behind non-adherence.

Thirdly, heavy physical labour during pregnancy was also perceived by participants as a potential cause of stillbirth. Some participants believed that engaging in strenuous tasks, such as carrying heavy loads, walking long distances or performing demanding household chores, could place undue stress on the body and negatively affect the baby. One woman recounted her experience of heavy household chores:

‘I was doing so many heavy duties here at home, like fetching firewood and water using a wheelbarrow. I was doing all those heavy household chores. I think they might have contributed.’ (P08, female, 32-years-old)

Fourthly, alcohol consumption during pregnancy was also perceived as a potential contributor to stillbirth. One male participant expressed suspicion that his partner may have consumed alcohol while pregnant, which he believed could have played a role in the outcome:

‘I would always advise my partner not to drink alcohol. I’m not saying she was drinking during the pregnancy; I’m just thinking that maybe her past drinking habits could have contributed. But I can’t say for sure whether she drank while she was pregnant or not. All I know is that I advised her not to, especially because she was pregnant.’ (P11, male, 24-years-old)

Lastly, participants identified the use of over-the-counter and traditional medicines to treat pregnancy-related sicknesses as a perceived cause of stillbirth. One participant highlighted concerns around the potential dangers of self-medicating during pregnancy, as illustrated:

‘Taking over-the-counter medications can be very dangerous, especially if you haven’t disclosed that you’re pregnant. Some of these medicines are harmful during pregnancy, so self-medicating is risky. Even when you’re in pain and someone suggests using herbs, that too can be dangerous, especially if you don’t know how strong the herb is or how it might affect you and your pregnancy.’ (P08, female, 32-years-old)

It was clear that the participant was concerned about the risks of self-medicating without professional guidance or non-disclosure of pregnancy status to a healthcare provider.

#### Emotional responses to stillbirth

The second theme at the individual level was emotional responses to stillbirth. Participants described a range of intense emotions following their loss, including shock, sadness, anger, uncertainty and helplessness. Reflecting on these emotional experiences, one participant who gave birth to twins at home shared:

‘The stillborn one was a twin; they were both boys. I gave birth to them here at home. When I looked at the two babies, I noticed that one was very small, and I wasn’t happy with how he looked. I could tell that something was wrong. I was confused at the time and didn’t fully understand what was happening, but I knew the other baby wasn’t alive based on how he appeared.’ (P01, female, 57-years-old)

During the interview, the participant expressed distress, confusion and sadness, especially in the moment of recognising that one of her babies was not alive. The statement, ‘I was confused at the time’, highlights the emotional shock and the lack of immediate understanding. Similarly, other participants expressed uncertainty, stating that they could not comprehend:

‘I don’t know how I reacted because I couldn’t comprehend what had happened; I was completely confused.’ (P10, female, 23-years-old)

‘I didn’t feel anything as I was numb. It didn’t hit me at that moment; I was still trying to make sense of what had happened.’ (P04, female, 39-years-old)

In addition to the feelings of sadness and uncertainties about stillbirth, participants described experiencing negative emotions triggered by painful memories, particularly when seeing other children who would have been the same age as their stillborn baby. As reflected in one participant’s account:

‘It feels so painful because I can’t help but keep counting. Whenever I see children who would be his age, it hurts. I always think to myself, “My child would be that age now if he had lived”. I even tell my daughter, who was born after him, that she was meant to have an older brother.’ (P06, female, 47-years-old)

Seeing other children of the same age as their baby would have, served as a constant and painful reminder of both their loss and the developmental milestones their child was never able to reach.

#### Self-doubt and shame

The third theme that emerged from the individual-level experiences was self-doubt and shame. Some participants questioned their ability to create or bring forth life following the stillbirth of their baby. The experience often led to feelings of personal failure, accompanied by embarrassment and internalised blame for not being able to deliver a living child. One male participant poignantly described this sense of self-doubt after experiencing a stillbirth:

‘When we had a stillbirth instead of a live baby, it was incredibly painful. I was ashamed, you know, because I used to brag that I was going to be a father. So, when things turned out the way they did, I became very frustrated and felt less of a man. Even though I said I had told my colleagues, I didn’t share exactly what had happened at first. I was ashamed. But when they asked more questions, I eventually told them the whole truth.’ (P09, male, 38-years-old)

The participant admitted to feeling ashamed, not only because of the loss itself but also because he had openly expressed excitement and pride about becoming a father. The stillbirth deeply affected his sense of identity, leading him to perceive himself as less of a man.

#### Fear of stillbirth recurrence

The last individual-level theme that emerged was the fear of experiencing another stillbirth in future pregnancies. A deep sense of fear about the possibility of experiencing another stillbirth was clearly expressed by one participant. This fear extended beyond emotional anxiety; it had a direct impact on their intimate life, as sexual activity was closely associated with the risk of pregnancy and, by extension, the potential for another loss. For this participant, the trauma of stillbirth created a lingering apprehension that affected both emotional well-being and physical relationships. This participant shared his fear of going through such a devastating experience again:

‘We’re even afraid of being intimate. She has this fear of what if she gets pregnant and something like this happens again?’ (P11, male, 24-years-old)

The participant expressed how the trauma of stillbirth created ongoing fear and emotional strain, deeply affecting intimacy and the couple’s relationship because of anxiety about the possibility of another loss.

### Interpersonal level factors

At the interpersonal level, the results reveal the role that family relationships, particularly with partners and family members, play in shaping individuals’ experiences of stillbirth. These relationships can serve as vital sources of emotional support, offering comfort and reassurance during a time of profound loss. However, they can also contribute to emotional distress, especially when responses from others are perceived as stigmatising. The quality of these family relationships strongly influences how individuals process and make sense of stillbirth.

#### Family relationships

Women emphasised the important role their spouses played in the aftermath of stillbirth, whether through their physical presence, involvement in funeral arrangements or the emotional support they provided during this difficult time. The following excerpts illustrate these experiences:

‘I received all the support I needed because the father of my child was always there for me, making sure everything was in order and that I had everything I needed during my pregnancy. Even when he was told that the baby had passed away, he was very hands-on; he even requested a car from work to help with the funeral preparations and to pick us up from the hospital.’ (P06, female, 47-years-old)

‘My husband was my strongest support system. I felt safe sharing my feelings and emotions about the loss with him. He listened, encouraged me, and was truly there for me.’ (P08, female, 32-years-old)

Both participants expressed strong appreciation for their partners’ support during and after the stillbirth. Despite these similarities, the nature of support described by the participants differs slightly. P06 focused on practical and logistical support, indicating how the partner ensured that her physical needs were met during pregnancy and took an active role in funeral arrangements. P08, on the contrary, emphasised emotional support and communication, describing how her husband created a safe space for her to express feelings and process the loss.

Beyond spousal support, family members, including aunts, mothers and sisters, also played a significant role in supporting parents after stillbirth, with their involvement deeply shaping how the loss was experienced and processed. The following excerpts illustrate these experiences:

‘My aunt played a very important role in supporting me. She told me that I had to accept what had happened because this kind of thing can happen to anyone. She also shared that she had experienced the loss of a child herself and encouraged me to accept everything so that I could move on with my life.’ (P04, female, 39-years-old)

‘I’ve been receiving a lot of support at home. My mom and sisters keep encouraging me, telling me that when the time is right, I will have a baby.’ (P10, female, 23-years-old)

While some participants’ experiences of stillbirth were shaped by positive and supportive family relationships, one participant reported having faced stigma and blame from family members, which compounded the emotional impact of the loss:

‘My first stillbirth was a very painful experience because we were living at the main family homestead at the time, where there was a first wife, a second wife, and many half-siblings. There were too many of us. I was treated very badly. They kept accusing me of killing my baby. I think they believed I had aborted the child, and they made so many hurtful remarks. It was deeply painful. They constantly reminded me of my child’s death. Eventually, I moved out and stayed with other people because I couldn’t bear to hear anything negative anymore. Honestly, I didn’t receive any real support after my first stillbirth, and I had no peace at all.’ (P02, female, 38-years-old)

It was clear that the participant’s experience of stillbirth was made more painful by the lack of family support and the presence of stigma and blame.

### Organisational level factors

At the organisational level, participants’ experiences of stillbirth were shaped by interactions with both religious institutions and the healthcare system. These two key structures played an important role in influencing how individuals processed their loss and the kind of support, or lack thereof, they received.

#### Religious institutions

Religious institutions, particularly churches, emerged as important sources of emotional, spiritual, physical and even financial support for participants who had experienced stillbirth. The following two excerpts illustrate these experiences:

‘We also received support from our church through prayers, and, as part of the church’s rules, they gave us a certain period of suspension [mourning period]. We were also supported financially, and, indeed, we received a good amount of support from the church.’ (P09, male, 38-years-old)

‘At church, they prepared a tea for me to drink to treat Rigoni [a condition believed to cause stillbirth]. Since then, I’ve never experienced it again. I’ve been fine, and all the other babies survived.’ (P01, female, 57-years-old)

The two participants highlighted the supportive role of religious institutions in the aftermath of stillbirth. Both participants described receiving help from their churches, one emphasising emotional, spiritual and financial support, while the other focused on spiritual healing through church remedies.

### Healthcare system

In addition to religious institutions, participants’ interactions with the healthcare system played a crucial role in shaping their experiences of stillbirth. The quality of care, communication, health education and emotional support provided by healthcare professionals influenced how parents experienced stillbirth. This sentiment was captured by a participant who remarked about health education provided by healthcare professionals:

‘They were teaching us everything […] how to breastfeed, how to care for yourself.’ (P10, female, 23-years-old)

Some participants described the quality of care provided by healthcare professionals as a positive aspect of their experience following stillbirth. This is reflected in the accounts of the following two participants:

‘Trust me, both the doctors and nurses did everything they could; they were running up and down, attending to me and trying their best. But still, I lost my baby. They explained that the baby had already passed stool inside my womb, so that’s what happened.’ (P05, female, 51-years-old)

‘The nurse told me that my baby wasn’t getting enough oxygen while inside the womb. After I gave birth, the nurse even showed me the baby and said, “You see, the baby isn’t breathing; your baby has passed away”.’ (P10, female, 23-years-old)

However, in contrast to some positive experiences, other participants pointed to serious gaps in communication and record-keeping within the healthcare system, which negatively affected their understanding of the stillbirth and their overall satisfaction with the care they received. This was illustrated by a participant who stated:

‘No, nothing was said to me. When someone gives birth, the nurses are supposed to record everything, including the time of birth or death, and any other important details. But nothing was documented. Even if the nurse may have written something down somewhere, maybe on a small piece of paper to be sent to the hospital, I never saw it.’ (P03, female, 28-years-old)

Moreover, some participants expressed concern about delays in receiving timely medical attention, which they believed may have contributed to the loss of their baby. One participant reflected that if healthcare workers had attended to her sooner, her baby might have survived:

‘The nurses took a long time to attend to me. They only checked my blood pressure with a cuff and said someone would come to fetch me, but I was bleeding. I waited for a very long time. I don’t know exactly how many minutes or hours, but I had arrived in the morning and wasn’t seen by a doctor until around noon. That’s when I was told my baby had passed away. I believe the baby died during that time I was left bleeding. I was deeply hurt because I truly believe that if they had attended to me earlier, maybe my baby wouldn’t have died. I arrived early, but they delayed.’ (P08, female, 32-years-old)

Furthermore, some participants described significant transport challenges in accessing healthcare facilities during critical moments. Delays in ambulance services and the need to hire private vehicles were commonly mentioned as barriers that hindered timely care. These transport-related difficulties were seen as contributing factors in the painful experience of stillbirth. The following excerpt illustrates this struggle as shared by two participants:

‘On that particular day, I started experiencing very strong pains. It wasn’t easy to get an ambulance to come to the village, so a car was hired to take me to the nearest clinic at [*name of clinic – confidential*]. When we arrived, they examined me and said they couldn’t detect the baby’s heartbeat. The healthcare workers then called an ambulance themselves, and I was transferred to [*name of hospital – confidential*]. By the time I delivered, the baby had passed away.’ (P05, female, 51-years-old)

‘By the time we finally arrived at the hospital, they examined me and told me that the baby was no longer alive; there was no heartbeat. They said the baby had likely died during the time I was bleeding at home.’ (P08, female, 32-years-old)

Both participants emphasised the devastating consequences of delayed access to emergency care, recounting how transport difficulties and extended periods of bleeding at home ultimately led to the heartbreaking news of their baby’s stillbirth.

### Societal level factors

At the societal level, participants’ experiences of stillbirth were shaped by prevailing cultural beliefs and social interpretations of pregnancy loss. One prominent belief that emerged was the concept of *Xirheti* in Xitsonga culture, which refers to the notion that a woman who experiences repeated stillbirths is incapable of carrying a baby to full term. This cultural belief not only influenced how stillbirth was understood but also impacted how affected women were perceived and treated within their communities. This is illustrated by one participant who stated:

‘When I was seven months pregnant, I gave birth, but the baby was stillborn. I knew it was early, as I hadn’t reached full term, but the baby didn’t survive and was buried. It was my first pregnancy, and I didn’t understand what had happened. Later, I became pregnant again, and at seven months, the same thing nearly happened; I almost lost the baby again. I was then taken to [name of place] for traditional treatment, where they said I had something called Xirheti, meaning I couldn’t carry a baby to full term. After receiving help from the traditional healer there, the baby survived. My third and fourth pregnancies also resulted in healthy births. However, during my fifth pregnancy, although I carried to full term, my baby again was stillborn.’ (P02, female, 38-years-old)

The participant appeared to grapple with a mix of confusion, hope and loss as she navigated the cultural beliefs of *Xirheti* in her pursuit of understanding, healing and the hope of becoming a successful mother.

### Policy level factors

At the policy level, participants’ experiences during pregnancy were influenced by the broader health guidelines and legal expectations surrounding maternal care in South Africa. In particular, several participants emphasised the importance of adhering to national antenatal care guidelines, which recommend regular clinic attendance to ensure early detection of complications. These policies were seen as critical for preventing adverse outcomes such as stillbirth. The following excerpts reflect participants’ awareness of and reflections on these guidelines:

‘The South African law is very clear; every pregnant woman is required to attend a clinic for antenatal care.’ (P01, female, 57-years-old)

‘When I became pregnant for the first time, I attended my clinic appointments on time.’ (P02, female, 38-years-old)

Both participants emphasised the importance of attending antenatal care, with P01 viewing it as a legal requirement in South Africa, while P02 reflected personal adherence to this guideline through timely clinic visits during pregnancy.

## Discussion

This study explored the experiences of parents who had experienced stillbirth, drawing on the socio-ecological framework to understand the multi-layered influences that shaped their perceptions and experiences of stillbirth. The findings highlight how stillbirth is not only a personal loss but also one that is shaped by broader interpersonal, institutional, societal and policy environments.

At the individual level, parents demonstrated varied understanding regarding the meaning of stillbirth and its causes. While some recognised biomedical explanations, such as foetal distress, others interpreted the experience through spiritual or supernatural lenses, including divine will and witchcraft. The varied understandings of stillbirth reflect a diverse range of perceptions and interpretations of what stillbirth means and its potential causes. These interpretations are shaped by personal, cultural and societal influences. Additionally, these perceptions align with earlier research emphasising the role of cultural and spiritual beliefs in shaping health outcomes.^11,12^ Emotional responses such as sadness, anger and fear of recurrence were prevalent, reflecting the psychological toll of stillbirth.^22,23^ For example, one participant’s experience of stillbirth not only triggered profound grief but also challenged their sense of identity and self-worth. The phrase ‘I felt less of a man’ reflects the internalisation of traditional gender roles, where the ability to father a living child is tied to perceptions of masculinity and competence. The findings highlight the importance of targeted mental health interventions, such as counselling services for parents who have experienced stillbirth, to address grief, self-doubt and emotional distress. While some participants had clear beliefs about stillbirth, others admitted to limited understanding, relying on healthcare professionals for explanations. This indicates a gap in health literacy and the need for effective communication from healthcare providers to address misconceptions and promote evidence-based knowledge.

At the interpersonal level, family and partner relationships emerged as critical factors influencing parents’ experiences of stillbirth. While some participants experienced positive familial and marital relationships, others faced stigmatisation and accusations, particularly from extended family members. One participant, for example, reported being accused of causing her baby’s death and feeling unwelcome in her own home. These contrasting experiences highlight how interpersonal relationships can serve as both a buffer against distress and a source of additional trauma, depending on the nature of the social support. This contrast highlights the need for family-centred interventions to educate family members about stillbirth and reduce stigma. Prior research corroborates the significance of spousal support in mitigating emotional distress and fostering resilience.^24^

Interactions with religious institutions and the healthcare system were central at the organisational level. Churches were often seen as sources of comfort, offering spiritual, emotional and even financial support. However, in some cases, religious practices contributed to feelings of guilt or restriction, particularly where stillbirth was framed as a spiritual failure. In the healthcare system, participants’ experiences were mixed. Some praised the compassionate care of doctors and nurses, while others described critical failures in communication, documentation and timely medical intervention. Delayed responses, a lack of explanation and limited follow-up care were noted as significant sources of distress. Gaps in communication and delays in medical attention echo existing literature on healthcare barriers in low-resource settings.^24,26,40^ These findings suggest a need for more consistent training in respectful maternity care and grief-sensitive communication among healthcare workers.

At the societal level, cultural beliefs played a central role in shaping the meaning attributed to stillbirth. Notably, the Xitsonga concept of *Xirheti*, which implies a woman’s inability to carry a pregnancy to term, emerged as a powerful narrative influencing how repeat stillbirths are understood. Such beliefs can contribute to stigma and self-blame, particularly in settings where cultural explanations overshadow biomedical understanding. Previous studies suggest that community-level interventions are particularly effective in enhancing access to care and reducing stigma.^41,42^ These findings illustrate how community norms and social narratives can deeply affect how parents process their grief and how they are treated within their social environments.

At the policy level, participants acknowledged the importance of adhering to national antenatal care guidelines, which emphasise regular clinic attendance as a means of identifying complications early. Some participants demonstrated awareness of these policies, linking timely clinic visits to the potential prevention of stillbirth. However, structural barriers such as transportation challenges and delayed ambulance services hindered access to care, especially in rural settings. These systemic issues suggest that while policies exist, their implementation remains uneven, and infrastructural gaps continue to limit their effectiveness.

## Study limitations

The findings of this study may be deeply rooted in specific Xitsonga cultural or religious beliefs, making it challenging to generalise the results to populations with different cultural perspectives on stillbirth and healthcare-seeking behaviour. In addition, the study likely provides a snapshot of participants’ experiences at a single point in time, without tracking their well-being over a longer period. A longitudinal approach could have provided deeper insights into the evolving nature of the experiences of parents following stillbirth. Addressing these limitations in subsequent studies, such as by including larger and more diverse samples, incorporating longitudinal designs and using mixed-method approaches, could provide a more comprehensive understanding of the complexities surrounding stillbirth and its impacts. Moreover, although the authors limited eligibility to those who had experienced a stillbirth within the past 5 years to reduce the potential for recall bias, they acknowledge that retrospective accounts are inherently subject to memory distortion or reinterpretation over time. Lastly, while data saturation was observed during analysis, evidenced by the repetition of key themes and the absence of new insights in later interviews, the authors acknowledge that a larger or more diverse sample might have yielded additional perspectives.

## Recommendations for interventions

To improve support for parents following a stillbirth, it is essential to implement accessible counselling and support groups that address grief, fear of recurrence and emotional trauma. Family-centred educational programmes should be developed to reduce stigma and foster understanding of stillbirth within cultural contexts. Strengthening healthcare systems is crucial, which includes enhancing patient-provider communication through training and addressing delays in medical attention by improving staffing and resources in facilities. Additionally, fostering partnerships between religious institutions and healthcare facilities can provide integrated spiritual and medical care, offering holistic support for grieving parents.

## Conclusion

Using the socio-ecological framework allowed for a nuanced understanding of the diverse and intersecting factors that shape the stillbirth experience. Addressing stillbirth requires a multi-level approach that includes individualised emotional support, strengthened family and community networks, compassionate healthcare practices, culturally sensitive public health messaging and more effective implementation of maternal health policies. Future interventions must be both context-specific and holistic, recognising that stillbirth is experienced not in isolation, but within a complex web of social, cultural and systemic influences. Future research should continue to explore the intersections of culture, healthcare access and stillbirth experiences to inform evidence-based interventions. By leveraging the socio-ecological framework, this study offers important considerations for improving the experiences of parents and reducing the burden of stillbirth.
